# Performance of DNA methylation analysis in the detection of high-grade cervical intraepithelial neoplasia or worse (CIN3+): a cross-sectional study

**DOI:** 10.1186/s13027-023-00555-2

**Published:** 2023-11-29

**Authors:** Yuxiang Liu, Yan Chen, Jing Xiong, Peng Zhu, Yuhang An, Shu Li, Puxiang Chen, Qing Li

**Affiliations:** 1 Department of Gynaecology and Obstetrics, The Second Xiangya Hospital, Central South University, 139 Renmin Road, Changsha, 410011 People’s Republic of China; 2 Department of Clinical Pharmacology, Xiangya Hospital, Central South University, 87 Xiangya Road, Changsha, 410008 People’s Republic of China; 3 Institute of Clinical Pharmacology, Central South University, Hunan Key Laboratory of Pharmacogenetics, 110 Xiangya Road, Changsha, 410078 People’s Republic of China; 4 Engineering Research Center of Applied Technology of Pharmacogenomics, Ministry of Education, 110 Xiangya Road, Changsha, 410078 People’s Republic of China; 5 Xiangya Medical Laboratory, Central South University, 110 Xiangya Road, Changsha, 410078 People’s Republic of China

**Keywords:** DNA methylation, hrHPV-positive, *SOX1*, CIN, Cervical cancer

## Abstract

It is commonly accepted that host genes show high methylation in cervical intraepithelial neoplasia 3 (CIN3) or worse (CIN3+). However, study quality varies, as does the clinical performance of markers in different populations. We aimed to validate candidate gene DNA methylation with standardized testing methods in the same batch of samples. We first compared the performance of 16 DNA methylation markers for detecting CIN3+ in the 82-sample training set, including 24 subjects with ≤ CIN1, 10 subjects with CIN2, 23 subjects with CIN3, and 25 subjects with cervical cancer (CC). Then five methylation markers were selected and subsequently validated among an independent set of 74 subjects, including 47 subjects with ≤ CIN1, 13 subjects with CIN2, 6 subjects with CIN3, and 8 subjects with CC. The results in the validation set revealed that methylation analysis of the *SOX1* (*SOX1*^*m*^) showed a superior level of clinical performance (AUC = 0.879; sensitivity = 85.7%; specificity = 90.0%). *SOX1*^*m*^ had better accuracy than cytology, with a reduced referral rate (23.0% vs. 31.4%) and a lower number of overtreatment (5 vs. 13) cases among high-risk human papillomavirus (hrHPV)-positive women. Importantly, among hrHPV-positive and *SOX1*^*m*^-negative women, only 1 CIN3 patient was at risk for follow-up after 1 year, whereas 1 CIN3 patient and 1 CC patient were at risk among hrHPV-positive and cytology-negative women. In this investigation, we screened 16 reported methylation markers to provide a basis for future studies related to potential precancerous lesion/cancer methylation markers in the Chinese population. The study also revealed that *SOX1*^*m*^ has optimal CIN3+ detection performance, suggesting that it may be a promising biomarker for detecting CIN3+ in the Chinese population.

**Supplementary Information:**

The online version contains supplementary material available at 10.1186/s13027-023-00555-2.

## Introduction

Cervical cancer (CC) had the fourth highest incidence and mortality among women worldwide in 2020 [1, 2], and its incidence had markedly increased in China since 2000 [3]. Most cervical cancers are in poor- and middle-income countries. Nevertheless, its incidence and mortality in the United States have dropped notably since the 1950s [4], as a result of prevention programs [5], which include human papillomavirus (HPV) vaccination (primary prevention) and screening (secondary prevention). Although prevention programs have grown greatly over the past [6], the measures have not been equitably implemented across and within countries [7]. As long as HPV vaccination is restricted, screening still serves as a cornerstone for detecting and preventing cervical cancer.

Over the past 30 years, research has shown a cause-and-effect relationship between high-risk HPV (hrHPV) infection of the cervix and cervical cancer, of which persistent hrHPV drives the slow progression of precancerous lesions and eventually cervical cancer [8]. Large longitudinal studies have found that hrHPV testing is effective as a primary screening tool [9, 10]. The current guidelines recommend hrHPV testing as the primary test or a co-test with cytology [11]. The hrHPV test as primary screening has a better sensitivity than cytology for detecting cervical intraepithelial neoplasia 3 (CIN3) or worse (CIN3+). However, hrHPV testing cannot distinguish whether the infection is transient or persistent, which results in less specificity, leading to unnecessary referrals to the gynecologist and anxiety in false-positive women. Cytology detecting cervical (pre)malignancies focuses on abnormal cells and has relatively limited sensitivity due to the subjectivity of analysis [12]. Cytology mitigates the above concerns through co-testing or triage of hrHPV infection patients, but the disadvantages of cytology testing still limit its use. Therefore, a high-accuracy and feasible triage strategy is urgently needed.

Aberrant DNA methylation patterns are a hallmark of cancer, and cytosine methylation (5mC) can lead to the activation of oncogenes and the inactivation of tumor suppressor genes, driving tumorigenesis. The methylation of CpG islands within gene promoter region is a frequently observed epigenetic phenomenon in many types of cancer, including cervical cancer [13]. The epigenetic alteration accumulated in epithelial cells is one of the processes underlying the driver of cervical carcinogenesis and progression. The literature [13–16] reported that promoter methylation levels of host genes, such as *EPB41L3*, *FAM19A4*, *JAM3*, *miR124-2*, *PAX1*, *ZNF671*, and *SOX1*, were related to the severity of cervical histological lesions. Methylation analysis could be a promising biomarker for the early detection of cervical lesions. However, the clinical representation of gene methylation varies in studies of various populations. Leeuwen et al. [17] evaluated the clinical performance of *EPB41L3* and JAM3 methylation (*EPB41L3*^*m*^ and *JAM3*^*m*^), showing that the sensitivities of *EPB41L3*^*m*^ and *JAM3*^*m*^ were 84% and 68%, respectively, for detecting CIN3+ in Slovenian patients. An exploratory study [18] was performed on Chinese patients, which also involved analyzing the performance of *EPB41L3*^*m*^ and *JAM3*^*m*^. The results indicated that the sensitivity (74.8%) of *EPB41L3*^*m*^ in detecting CIN3+ was poorer than that in the Slovenian population, but the sensitivity (80.4%) of *JAM3*^*m*^ was superior. Therefore, it is necessary to evaluate the clinical performance of potential candidate genes in the same cohort.

Although various methylation biomarker-based kits [19–22] were dedicated to detecting CIN3+ in HPV-positive women, we could still keep optimizing the methylation marker for clinical implementation [23] in the Chinese population. We surveyed a variety of publications and found that most DNA methylation biomarker-based studies had been performed by distinct research groups using different analysis methods in different populations. Therefore, in the current study, we sought to evaluate the clinical performance of candidate gene DNA methylation in the same batch of clinical samples. The performance of the selected methylation marker was subsequently compared to that of cytology in hrHPV-positive women.

## Methods

### Clinical specimens

The clinical study was approved by the local medical ethics review committee. Subjects enrolled in methylation testing at Xiangya Hospital and Second Xiangya Hospital from January 2021 to June 2022. Study inclusion criteria included cervical screening populations, or who were suspected of cervical lesions on gynecological examination. Patients with any history of CIN or cervical cancer treatment, a current pregnancy, or menstruation were excluded. All subjects had the colposcopic examination results. Cervical biopsies were collected from each visible lesion for histological evaluation and categorized in accordance with international criteria including ≤ CIN1, CIN2, CIN3 (including carcinoma in situ), or CC. To ensure the quality of the diagnosis, two experienced pathologists independently reviewed the histology slides. Cytology was categorized as negative for intraepithelial lesion or malignancy (NILM), atypical squamous cells of undetermined significance (ASC-US), low-grade squamous intraepithelial lesion (LSIL), atypical squamous cells: cannot exclude high-grade squamous intraepithelial lesion (ASC-H) and high-grade squamous intraepithelial lesion (HSIL). The study enrolled 156 women, 82 of whom were trained for screening candidate genes, and 74 of whom were collected for validation of selected genes with the complete information (with cytology and hrHPV).

### Candidate gene selection

Candidate methylation genes were selected according to the following criteria: (1) the genes were reported in previous studies as DNA markers to identify cervical lesions; (2) primer sequences were shown in the literature or were designed to stabilize detection in our experiment. Finally, *GFRA1*, *MIR124-2*, *ASCL1*, *CCDC181*, *EPB41L3*, *JAM3*, *PAX1*, *SORCS1*, *PCDHA13*, *LOC100289333*, *BOLL*, *FAM19A4*, *MIR129-2*, *ZIC1*, *SOX1 and SST* were selected as candidate genes [13–15, 24–26].

### Sample preparation and hrHPV testing

The clinician took cervical scrapes using a cervix brush and directly placed the brush in the preservation solution (ThinPrep, MA). The hrHPV test was conducted by a fluorescence quantitative PCR (ABI 7500) system (Life Tech, USA) with an hrHPV kit (Sansure-Biotech, China). The kit uses type-specific probes to detect pooled results for hrHPV types, including HPV 16, 18 and other types (31, 33, 35, 39, 45, 51–53, 56, 58–59, 66 and 68).

### Sodium bisulfite treatment and methylation testing

DNA from residual cervical scrapes was extracted with a HiPure Universal DNA Kit (Magen Biotech, China) after analysis of cytology and hrHPV, and the concentrations and 260/280 ratios were determined using a Nanodrop microspectrophotometer (Thermo Fisher Scientific, USA). Bisulfite conversion was performed on the isolated DNA with an EZ DNA Methylation Kit (Zymo Research, USA). DNA was purified and then eluted with 20 μL solution. All procedures were performed in accordance with the manufacturer’s instructions. Methylation analysis of the candidate genes and internal reference gene (*ACTB*) were evaluated using SYBR Green I (Solarbio, China) and the ABI 7500 system. PCRs were conducted with 5 μL of real-time PCR mix, 1 μL of bisulfite-treated DNA, primers for the respective genes and nuclease-free water to a final volume of 10 μL (Additional file 1: Table S1). The PCR conditions were as follows: 95 °C for 5 min, 45 cycles at 95 °C for 15 s and 60 °C for 30 s, and a standard melting curve. To ensure the quality of the samples, the *ACTB* cycle threshold (Ct) of all samples should be below 30. The delta cycle threshold value (ΔCt) of each sample was calculated by the candidate methylated gene Ct minus the methylated *ATCB* gene Ct. Nonbisulfite-converted gDNA was used as a negative control, and bisulfite-converted DNA from women with cervical cancer was used as a positive control for each MSP plate.

### Statistical analysis

The cutoff values of each gene DNA methylation were generated from methylated gene ΔCt of all samples with a receiver operating characteristic curve (ROC). The methylated genes with maximal values of the Youden index were determined as the optimal cutoff values. The optimal cutoff values in the training set were generated from 82 subjects, while the optimal cutoff values in the validation set were generated from 156 subjects. The area under the ROC curve (AUC) differentiating between CIN2- (≤ CIN1 and CIN2) and CIN3+ (CIN3 and CC) was greater than or equal to 0.8 (AUC ≥ 0.8). The positivity of cytology, hrHPV or methylation testing was calculated according to the cervical disease status confirmed by histology. Clinical sensitivity and specificity were estimated along with the exact 95% confidence interval (CI), which was the proportion calculated assuming a binomial distribution. Women with positive results in both hrHPV and DNA methylation testing were classified as triage positive and others were classified as triage negative. All analyses were conducted by using Statistical Product and Service Solutions (SPSS) Statistics 21.0 software (IBM Corporation, USA).

## Results

### Candidate DNA methylation markers in the training set

**Fig. 1:**
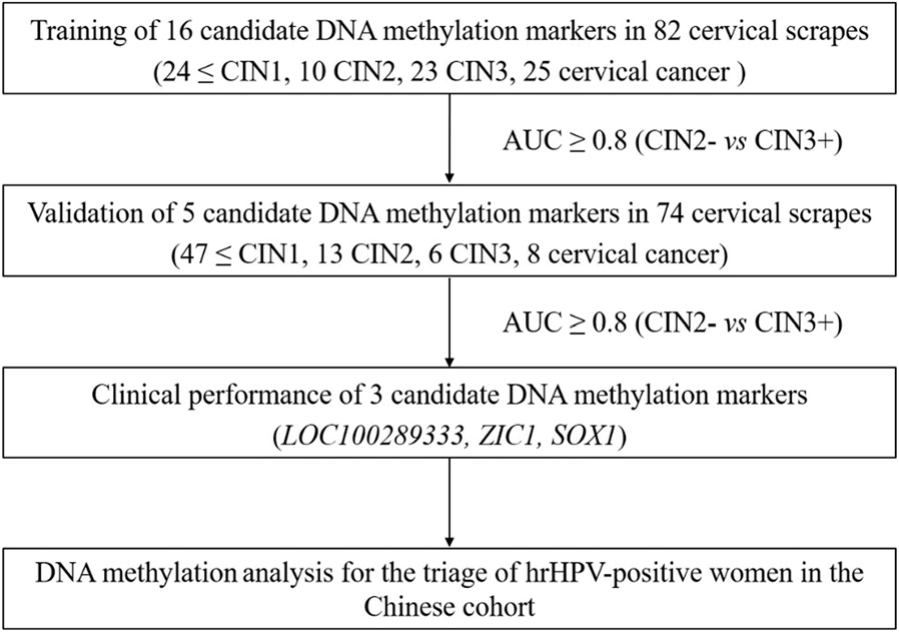
Flow scheme for the identification of the CIN3+ methylation marker. AUC, area under the curve; hrHPV, high-risk HPV

**Fig. 2:**
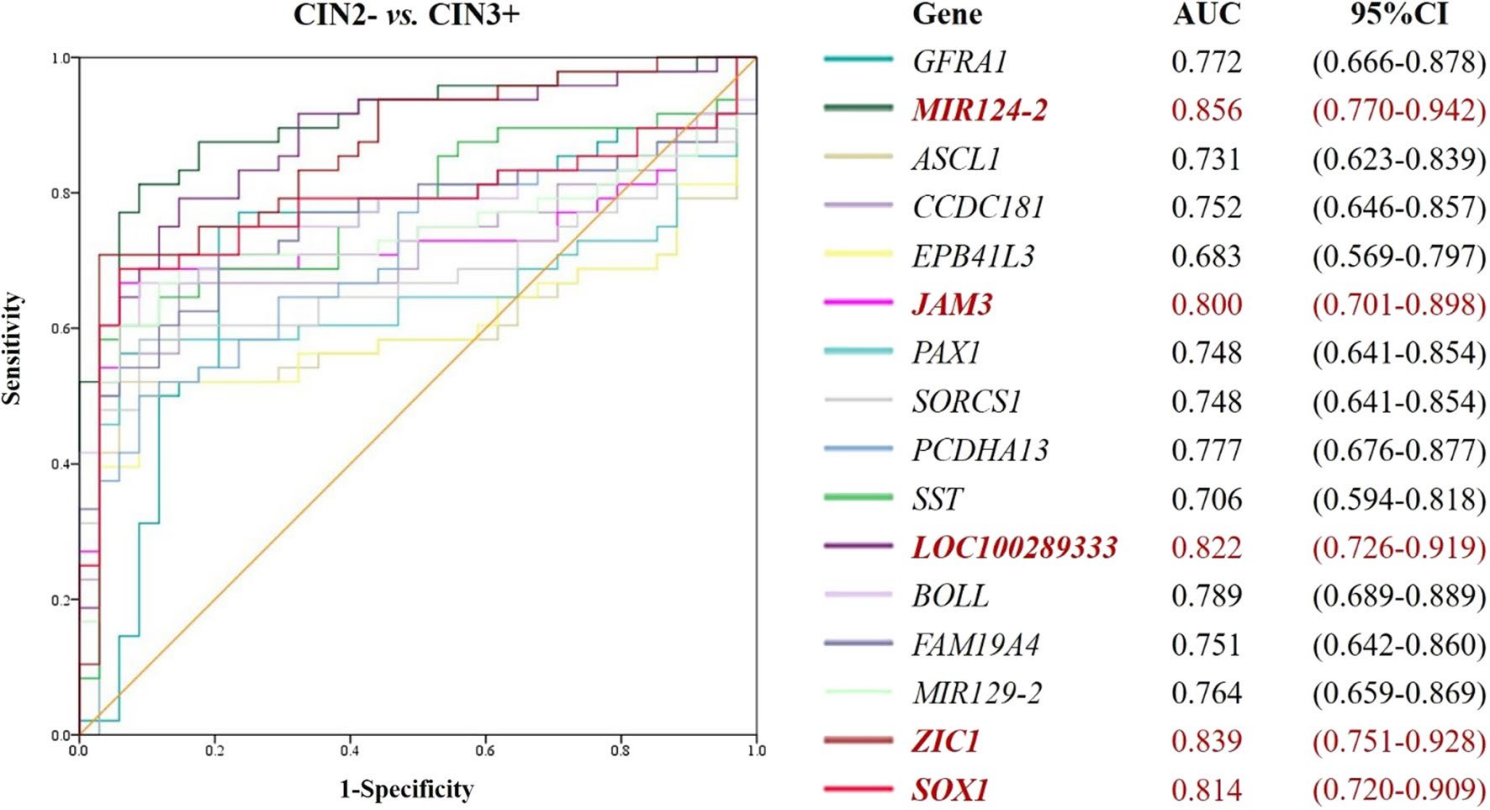
ROC curves and corresponding AUCs of candidate gene DNA methylation markers differentiated between CIN2- and CIN3+ detection. Power the methylation of 16 candidate genes in differentiating CIN3+ from CIN2- subjects. AUC, area under the curve; CI, confidence interval

A flow scheme of the study is shown in Fig. 1. Pathology results for 82 samples were as follows: ≤ CIN1, n = 24 (29.3%); CIN2, n = 10 (12.2%); CIN3, n = 23 (28.0%); and CC, n = 25 (30.5%). DNA isolated from 82 cervical scrapes was bisulfite-treated and tested in MSP experiments for the 16 candidate DNA methylation markers *GFRA1*, *MIR124-2*, *ASCL1*, *CCDC181*, *EPB41L3*, *JAM3*, *PAX1*, *SORCS1*, *PCDHA13*, *LOC100289333*, *BOLL*, *FAM19A4*, *MIR129-2*, *ZIC1*, *SOX1 and SST*. There were 5 genes with AUCs greater than or equal to 0.8 (AUC ≥ 0.8) showing a distinction between CIN2- and CIN3+ in cervical scrapings (Fig. 2), which were validated next. The AUCs of *MIR124-2*, *JAM3*, *LOC100289333*, *ZIC1*, and *SOX1* were 0.856 (95% CI 0.770–0.942), 0.800 (95% CI 0.701–0.898), 0.822 (95% CI 0.726–0.919), 0.839 (95% CI 0.751–0.928) and 0.814 (95% CI 0.720–0.909), respectively. The cutoff values corresponding to the above five genes were 9.89, 9.22, 6.2, 3.29 and 5.25, respectively (Additional file 1: Table S2). The positive rates of 16 candidate genes in the training set are shown in Additional file 1: Fig. S1. Eleven genes were excluded from further analysis.

### Patients and histological outcomes

The clinicopathological data and cytology results of the validation cervical scrapes are summarized in Table 1. Pathology results for 74 samples were as follows: ≤ CIN1, n = 47 (63.5%); CIN2, n = 13 (17.6%); CIN3, n = 6 (8.1%); and CC, n = 8 (10.8%). CIN3+ subjects (average age 54.3 years) were older than CIN2- subjects (average age 43.0 years) (*p* < 0.05). The cytology results included 51 (68.9%) cases of NILM, 8 (10.8%) cases of ASC-US, 1 (1.4%) case of LSIL, 3 (4.1%) cases of ASC-H, and 11 (14.9%) cases of HSIL. Cytological results showed that NILM constituted 86.3% of ≤ CIN1 and ASC-US constituted 62.5% of CIN2, while HISL constituted 45.4% of cancer.

**Table 1:** Clinicopathological data of validation samples

	≤ CIN1	CIN2	CIN3	CC	Total
Number of subjects					
N (%)	47(63.5)	13(17.6)	6(8.1)	8(10.8)	74
Age					
Mean ± SD Range	44.6 ± 9.8 26.0–72.0	37.2 ± 10.3 19.0–57.0	52.0 ± 12.9 38.0–67.0	56.0 ± 7.7 47.0–68.0	45.1 ± 11.1 26.0–72.0
Cytology results					
NILM (%) ASC-US (%) LSIL (%) ASC-H (%) HSIL (%)	44(86.3) 0 0 1(33.3) 2(18.2)	3(5.9) 5(62.5) 1(100.0) 2(66.7) 2(18.2)	2(3.9) 2(25.0) 0 0 2(18.2)	2(3.9) 1(12.5) 0 0 5(45.4)	51 8 1 3 11

### Clinical performance of five DNA methylation markers in the validation set

**Fig. 3:**
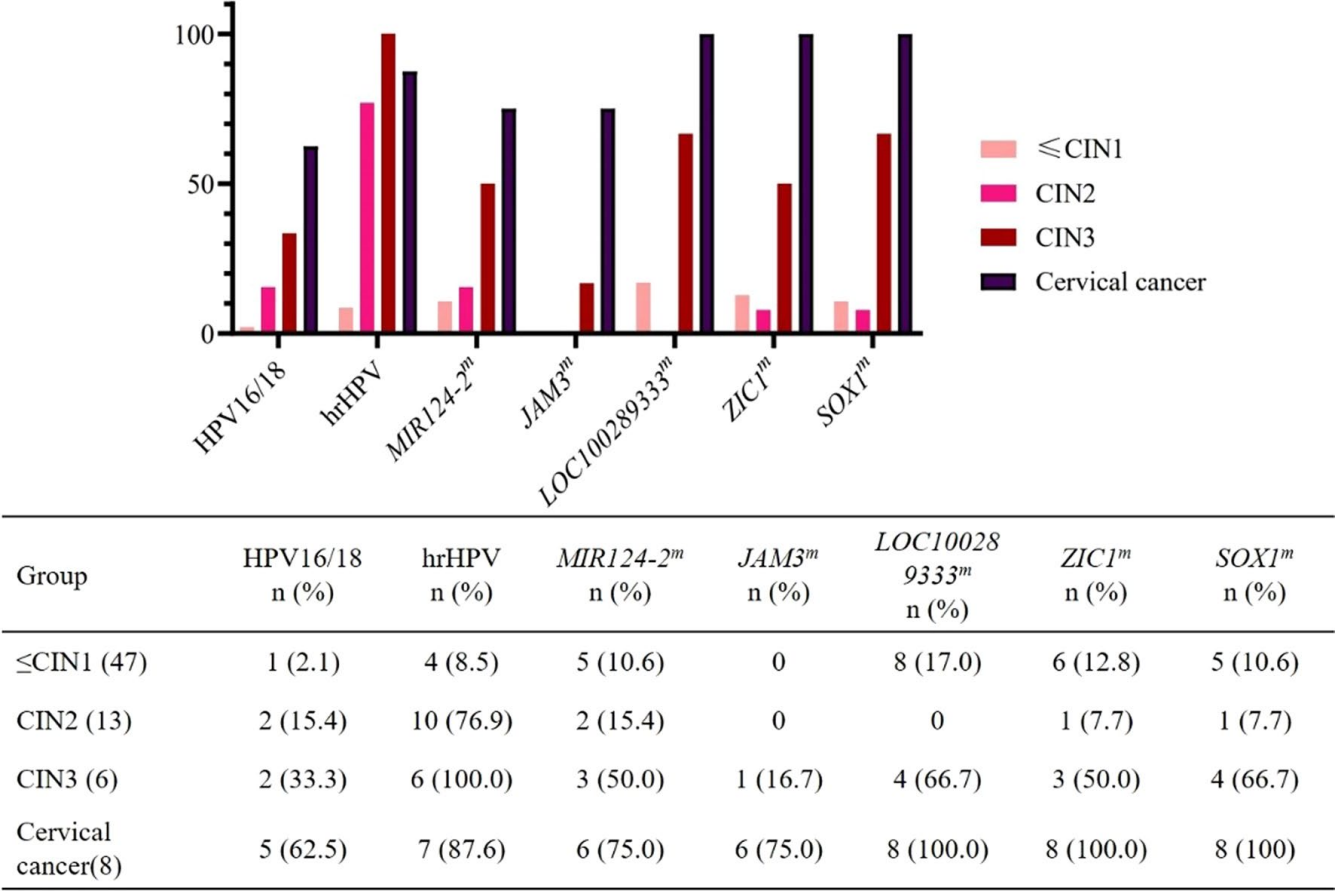
hrHPV positivity and methylation positivity of five genes in cervical scrapings (n = 74). The distribution of the methylation positivity of the five selected markers in the ≤ CIN1, CIN2, CIN3, and CC groups

Five genes selected from the training set were validated with 74 cervical scrapings (Fig. 3). The positivity rate of HPV16/18 and methylation raised with increased pathological grade. Compared to the results of cytology (sensitivity: 71.4%, 95% CI 42.0–90.4%; specificity: 78.3%, 95% CI 65.4–87.5%), the sensitivity of hrHPV (92.9%, 95% CI 64.2–99.6%) was significantly higher with a slightly lower specificity (76.7%, 95% CI 63.7–86.2%), and the sensitivity of HPV16/18 genotyping (50.0%, 95% CI 24.0–76.0%) was lower with a higher specificity (95.0%, 95% CI 85.2–98.7%). Overall, methylation rates ranged from 16.7% to 66.7% for CIN3 and from 75.0% to 100.0% for cervical cancer. Single methylation of *LOC100289333* (*LOC100289333*^*m*^), *ZIC1* (*ZIC1*^*m*^) and *SOX1* (*SOX1*^*m*^) tested positive in all cervical cancer scrapings. The new cutoff values in the validation set were recalculated from 156 subjects. The AUCs of *LOC100289333*^*m*^, *ZIC1*^*m*^, and *SOX1*^*m*^ that distinguished CIN2- and CIN3+ in the validation set were still greater than 0.8, with 0.862 (95% CI 0.744–0.980), 0.835 (95% CI 0.700–0.969) and 0.879 (95% CI 0.763–0.994) by the new cutoff values of 6.27, 3.29 and 5.25, respectively. The AUCs of *MIR124-2*^*m*^ and *JAM3*^*m*^ were less than 0.8, with 0.763 (95% CI 0.605–0.922) and 0.750 (95% CI 0.576–0.924), respectively, resulting in exclusion from further analysis (Table 2). *LOC100289333*^*m*^ and *SOX1*^*m*^, compared with *ZIC1*^*m*^ (78.6%, 95% CI 48.8–94.3%), had better clinical performance, and which sensitivity was greater than 85% (85.7%, 95% CI 56.2–97.5%). However, *SOX1*^*m*^ had higher specificity (90.0%, 95% CI 78.8–95.9%) than *LOC100289333*^*m*^ (86.7%, 95% CI 74.9–93.7%).

**Table 2:** Sensitivity and specificity of cytology, hrHPV, HPV16/18 genotyping and DNA methylation for detecting CIN3+

	Sensitivity	Specificity	AUC^b^
	95% CI	95% CI	95% CI
Cytology	71.4% (42.0–90.4%)	78.3% (65.4–87.5%)	0.749 (0.598–0.900)
hrHPV	92.9% (64.2–99.6%)	76.7% (63.7–86.2%)	0.848 (0.744–0.952)
HPV16/18	50.0% (24.0–76.0%)	95.0% (85.2–98.7%)	0.725 (0.55–0.898)
Methylation markers^a^			
*miR124-2*^*m*^ *JAM3*^*m*^ *LOC100289333*^*m*^ *ZIC1*^*m*^ *SOX1*^*m*^	64.3% (35.6–86.0%) 50.0% (24.0–76.0%) 85.7% (56.2–97.5%) 78.6% (48.8–94.3%) 85.7% (56.2–97.5%)	88.3% (76.8–94.8%) 100.0% (92.5–100.0%) 86.7% (74.9–93.7%) 88.3% (76.8–94.8%) 90.0% (78.8–95.9%)	0.763 (0.605–0.922) 0.750 (0.576–0.924) 0.862 (0.744–0.980) 0.835 (0.700–0.969) 0.879 (0.763–0.994)

^a^Using a threshold for positivity at a methylation ratio of 8.56 for *MIR124-2*, 5.94 for *JAM3*, 6.27 for *LOC100289333*, 3.29 for *ZIC1*, and 5.25 for *SOX1*

^b^The performance of each marker in cervical scrapes was evaluated by AUC with 95% (CI)

### DNA methylation markers for the triage of hrHPV-positive women

**Fig. 4:**
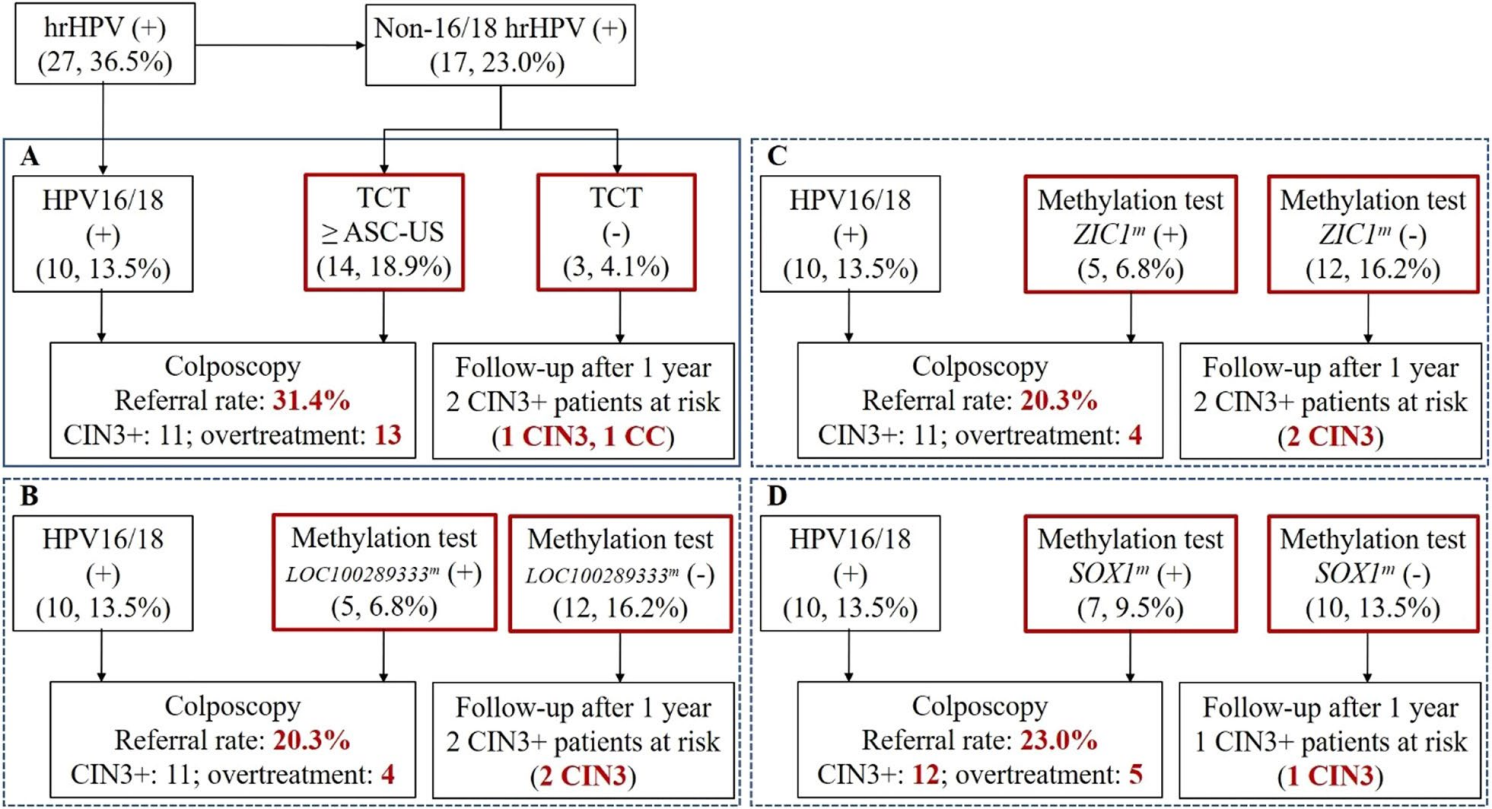
Cytology and methylation tests for the triage and management of hrHPV-positive women in the Chinese cohort. Outcomes of the triage chart are shown for TCT (**A**), *LOC100289333*^*m*^ (**B**), *ZIC1*^*m*^ (**C**), and *SOX1*^*m*^ (**D**). TCT, Thinprep liquid-based cytology test

Forty-four of 74 (59.5%) subjects were hrHPV positive, with HPV16/18 positivity constituting 36.5% and non-16/18 hrHPV positivity constituting 23.0% of all subjects (Fig. 4). HPV16/18 genotyping was used to triage hrHPV-positive scrapings, and subjects with HPV16/18 positivity were referred directly to colposcopy. Among HPV16/18-positive women, 8 cases of CIN3+ (3 cases CIN3 and 5 cases CC) were detected, and 3 cases of CIN2- (1 case CIN1 and 2 cases CIN2) were overtreated. The rate of referral for colposcopy was 18.9% in non-16/18 hrHPV (+) plus cytology (≥ ASC-US) subjects. Thus, when cytology was applied as a screening strategy for hrHPV (+), the overall rate of referral for colposcopy was 31.4%, at which point the number of overtreatments was 13, and the number of missed diagnoses was 1 CIN3 and 1 CC (Fig. 4A). *LOC100289333*^*m*^, *ZIC1*^*m*^, or *SOX1*^*m*^ in hrHPV-positive women was evaluated, with colposcopy referral rates of 20.3%, 20.3% or 23.0% and missed diagnoses in subjects with two, two or one CIN3 cases (Fig. 4B, D).

## Discussion

In the cross-sectional study, we assessed the performance of 16 possible methylation markers by standardized testing methods in the same batch of samples. We found *SOX1*^*m*^ to be a significant biomarker for detecting CIN3+ subjects. The data showed that *SOX1*^*m*^ was superior to cytology in hrHPV-positive women. The current results imply that *SOX1*^*m*^ analysis identifying cervical lesions is promising in the Chinese population.

To solve the low specificities of hrHPV testing, cytology is used for effective triage and management of hrHPV-positive samples. However, for hrHPV screening, only districts with high-quality cytology can relatively balance detection and over-referrals. It is challenging to recruit and relent skilled cytologists in rural areas. Methylation analysis with high reproducibility and objectivity is a feasible alternative biomarker [27]. As previously described, host gene DNA methylation might be useful for the triage and management of hrHPV-positive women. Compared to cytology, *LOC100289333*^*m*^, *ZIC1*^*m*^, or *SOX1*^*m*^ had better accuracy for triage and management of hrHPV-positive women (Fig. 4), with lower colposcopy referral rates along with improved CIN3+ case detection. Moreover, all cancer patients were identified as hrHPV-positive women.

*LOC100289333* is a pseudogene, whose methylation analysis is first reported in our study. Our data revealed that *LOC100289333*^*m*^ has a middle level of performance among the three validated genes in the detection of CIN3+ with a sensitivity of 85.7% (56.2–97.5%) and specificity of 86.7% (74.9–93.7%). However, the performance in hrHPV-positive women was less favorable with a sensitivity of 66.7% (24.2–94.0%) and specificity of 90.9% (57.1–99.5%), which may be due to quantity bias from the small population size. The ZIC1 protein was first studied in cerebellum tissues and serves as a transcription factor in the central nervous system, muscle, and bone growth and development. Recent studies [28] found that the levels of *ZIC1* mRNA and protein in cervical cancer and increased CIN grade were significantly decreased compared with normal and CIN samples, which is presumably a promising biomarker for prognosis. In addition, *ZIC1*^*m*^ was elevated when the lesions of the cervix worsened [25, 29], which correlated with the downregulation of *ZIC1* in increased lesion grades. *ZIC1*^*m*^ in cervical scrapes was associated with the presence and progression of lesions among hrHPV-positive women, with a clinical performance of sensitivity of 86.3%, specificity of 80.4%, and a highest AUC of 0.89 [30]. In a Dutch cohort, Verhoef et al. [25] reported that *ASCL1*^*m*^, with the highest AUC (0.844), showed better performance than *ZIC1*^m^ (AUC = 0.725) and *SST*^*m*^ (AUC = 0.720) for CIN3+ detection among candidate methylation markers. In our data (Fig. 2 and Additional file 1: Table S3), the performance of *ZIC1*^*m*^ in discerning CIN3+ in hrHPV-positive women was better than that of *ASCL1*^*m*^. Use of *ZIC1*^m^ in hrHPV-positive women could decrease colposcopy referral rates (31.4% vs. 20.3%) compared with cytology and avoid missed diagnoses of cancer subjects.

The SOX1 protein is important in developmental processes as a transcription factor. The study showed that SOX1 might be a tumor suppressor in cervical cancer partly through the Wnt/β-catenin signaling pathway [31]. Furthermore, the expression level of *SOX1* was higher in the normal groups than in the CC and CIN groups [32]. Hypermethylation of *SOX1*, resulting in decreased expression, was recognized as a potential biomarker for high-grade lesions. In the present study, *SOX1*^*m*^ had a specificity of 81.8% in hrHPV‐positive scrapings, detecting CIN3+ (Additional file 1: Table S3) with a similar sensitivity (83%), which was better than the specificities previously published (74%) [26]. Compared to the results reported previously, the performance of *SOX1*^*m*^ in detecting CIN3+ (sensitivity: 83.3% and specificity: 81.8%) in our data was better than that of the two commercial methylation-specific PCR assays [21] (GynTect®: sensitivity: 66.7% and specificity: 84.1%; QIAsure methylation test: sensitivity: 78.6% and specificity: 68.2%) in hrHPV‐positive scrapings and poorer than that of GynTect® (sensitivity: 94.1% and specificity: 68.4%) performed at three rural sites in China [22]. In comparison to the Slovenian population, high sensitivity of *SOX1*^*m*^ was observed among methylation markers for CIN3+ instead of *EPB41L3*^*m*^ in our study. Although our goal was to evaluate the biomarker potential of candidate gene methylation to triage hrHPV-positive women, we also observed elevated *SOX1*^*m*^ in a hrHPV-negative woman (Additional file 1: Fig. S2). We used 14 cases of CIN3+ and 60 cases of CIN2- for the calculation. Notably, all 8 cancer cases were detected by *SOX1*^*m*^. This suggests that analysis of methylation may help identify women at risk of developing cervical cancer regardless of hrHPV status.

In addition to cervical scrapes, DNA methylation analysis in urine is also feasible. Many women felt unpleasant after experiencing the collection of cervical scrapes. Urine as a kind of noninvasive sample including cervicovaginal secretions, is easily accepted by patients, offering an effective solution to attract non-responders. The clinical performance of *ZIC*^*m*^ discerning CIN3+ from CIN2- in scrapes was higher than that in urine (AUC = 0.558, 95% CI 0.400–0.742) [33], whereas methylation analysis of *SOX1* was not yet reported, which could be considered in future trials.

The strength of our study is the analysis of the DNA methylation of sixteen candidate genes with MSP in the same batch of clinical samples. Despite the expense of whole genome methylation sequencing, different ethnic groups should be taken into account before translating the available results to the clinic. Our findings underscore the essentiality of verifying methylation biomarkers in different nationalities or populations. The study is cross-sectional without follow-up, which is a major limitation. It is necessary to ascertain the optimal reassessment interval for hrHPV-positive women who test negative for *SOX1*^*m*^ by longitudinal evaluation. Another limitation of this study is quantity bias due to the small sample size. Although subjects were collected randomly from women who met the criteria, the population in the methylation test may not represent all women. Our study focuses on CIN3+ rather than CIN2+ to evaluate clinical performance. It is moderately reproducible when women are diagnosed with CIN2. In addition, young CIN2 patients have a relatively high regression rate [34]. The CONCERVE study [19] suggested that women with untreated CIN2/3 and negative methylation results showed clinical regression. This might reveal that excessive improvement of methylation test performance in detecting CIN2+ without other combination test strategies could result in excessive attention and considerable overtreatment.

## Conclusion

The performance of the candidate methylation markers was evaluated in the same batch of samples, which could provide a basis for future studies related to potential precancerous lesion/cancer methylation markers in the Chinese population. The data of our study show that the performance of *SOX1*^*m*^ has a high sensitivity among candidate methylation markers for CIN3+ in the Chinese cohort. Meanwhile, it is a promising biomarker of triage in hrHPV-positive women for colposcopy referral. Further studies are still warranted in screening populations.

### Supplementary Information

**Additional file 1: Table S1.** Primer sequence of 16 candidate methylation genes.** Table S2.** Sensitivity, specificity, and cut-off of candidate gene DNA methylation markers differentiated between CIN2- and CIN3+ detection.** Fig. S1.** Sixteen genes methylation positivity in cervical scrapings (n = 82). The distribution of the methylation positivity of the 16 candidate markers in ≤CIN1, CIN2, CIN3, and CC group.** Table S3.** Sensitivity and specificity of cytology and DNA methylation for detecting CIN3+ in hrHPV-positive women.** Fig. S2.*** SOX1*^*m*^ analysis in the Chinese cohort.

## Data Availability

The datasets used and analyzed in the current study are available where appropriate from the corresponding author on reasonable request.

## Abbreviations

ASCL1: Achaete-scute family bHLH transcription factor 1
ASCUS: Atypical squamous cells of undetermined significance
AUC: The area under the curve
BOLL: Boule homolog, RNA binding protein
CC: Cervical cancer
CCDC181: Coiled-coil domain containing 181
CI: Confidence interval
CIN2: Cervical intraepithelial neoplasia grade 2
CIN3: Cervical intraepithelial neoplasia grade 3
Ct: Cycle threshold
FAM19A4: Family with sequence similarity 19 (chemokine (C–C motif)-like) member A4
gDNA: Genomic DNA
GFRA1: GDNF family receptor alpha 1
HPV: Human papillomaviruses
hrHPV: High-risk human papillomavirus
JAM3: Junctional adhesion molecule 3
LSIL: Low-grade squamous intraepithelial lesion
MIR124-2: MicroRNA 124-2
miR129-2: MicroRNA 129-2
MSP: Methylation-specific PCR
PAX1: Paired box gene 1
PCDHA13: Protocadherin alpha 13
ROC: Receiver operating characteristic
SORCS1: Sortilin related VPS10 domain containing receptor 1
SOX1: SRY-box transcription factor 1
TCT: Thinprep cytologic test
ZIC1: Zinc finger protein of the cerebellum 1
