# Supplementary material for: Performance of DNA methylation analysis in the detection of high-grade cervical intraepithelial neoplasia or worse (CIN3+): a cross-sectional study
Source: Infect Agent Cancer. 2023 Nov 29;18:77. doi: 10.1186/s13027-023-00555-2 (PMC10687787; doi:10.1186/s13027-023-00555-2)
Supplement: Supplementary file 1 — Additional file 1: Table S1. Primer sequence of 16 candidate methylation genes. Table S2. Sensitivity, specificity, and cut-off of candidate gene DNA methylation markers differentiated between CIN2- and CIN3+ detection. Fig. S1. Sixteen genes methylation positivity in cervical scrapings (n = 82). The distribution of the methylation positivity of the 16 candidate markers in ≤CIN1, CIN2, CIN3, and CC group. Table S3. Sensitivity and specificity of cytology and DNA methylation for detecting CIN3+ in hrHPV-positive women. Fig. S2. SOX1m analysis in the Chinese cohort. [file 13027_2023_555_MOESM1_ESM.docx]

**Table. S1 primer sequence of 16 candidate methylation genes.**

| Gene | Forward primer | Reverse primer |
| --- | --- | --- |
| *GFRA1* | GGGTTTAATTCGAGACGTTGAAC | CCACCGAAAACTCGAACTCC |
| *MIR124-2* | TGGTGATGGAGGAGGTTTAGTAAGT | CGTAAAAATATAAACGATACGTATACCTACGT |
| *ASCL1* | GTCGTAGCGGTAGCGTAGAGC | CGTTTACAACGCATCAATTCG |
| *CCDC181* | TTGAGGATATTGTATGCGTTTGC | GACACCTACCTCCGCGACA |
| *EPB41L3* | GGGATAGTGGGGTTGACGC | ATAAAAATCCCGACGAACGA |
| *JAM3* | GGGATTATAAGTCGCGTCGC | CGAACGCAAAACCGAAATCG |
| *PAX1* | TATTTTGGGTTTGGGGTCGC | CCCGAAAACCGAAAACCG |
| *SORCS1* | GGTGCGTAGCGTAGTTCGGTC | CCAAATTACGCCGCGATAAA |
| *PCDHA13* | CGTTTTCGAGGAAGTAAAATACGG | GTATCTTTTAAACGCCACCCGAA |
| *LOC100289333* | GTTTCGGTTTTAGTTTCGGCG | CACTACTCCTCCCACGCACG |
| *BOLL* | GAGACGTTTCGGTTCGGAGTT | CGTCGCCTCGAATAACAAATAA |
| *FAM19A4* | AGTCGGGCGGTTCGGTTA | CGTAAAAATATAAACGATACGTATACCTACGT |
| *MIR129-2* | CGGCGGCGAATCGAAG | GAATACGCCCTCCGCAAATAC |
| *ZIC1* | TCGGGTTTTACGAGTAGGTTGTC | GCGAACTAATCACCTAACCGTACTA |
| *SOX1* | TTGTAGTTTTCGAGTTGGAGGTC | AAAACGATACGCTAAACCCG |
| *ACTB* | TGGTGATGGAGGAGGTTTAGTAAGT | TGGTGATGGAGGAGGTTTAGTAAGT |

**Table. S2. Sensitivity, specificity, and cut-off of candidate gene DNA methylation markers differentiated between CIN2- and CIN3+ detection.**

| Gene | sensitivity |  | specificity |  | Cut-off |
| --- | --- | --- | --- | --- | --- |
|  | 95% CI |  | 95% CI |  |  |
| *GFRA1* | 75.0% (60.1-85.9%) |  | 79.4% (61.6-90.7%) |  | 10.94 |
| *MIR124-2* | 77.1% (62.3-87.5%) |  | 94.1% (78.9-99.0%) |  | 9.89 |
| *ASCL1* | 52.1% (37.4-66.5%) |  | 94.1% (78.9-99.0%) |  | 10.93 |
| *CCDC181* | 56.3% (41.3-70.2%) |  | 94.1% (78.9-99.0%) |  | 4.45 |
| *EPB41L3* | 39.6% (26.1-54.7%) |  | 97.1% (82.9-99.8%) |  | 4.99 |
| *JAM3* | 68.8% (53.6-80.9%) |  | 91.2% (75.2-97.7%) |  | 9.22 |
| *PAX1* | 58.3% (43.3-72.1%) |  | 91.2% (75.2-97.7%) |  | 8.69 |
| *SORCS1* | 58.3% (43.3-72.1%) |  | 91.2% (75.2-97.7%) |  | 9.37 |
| *PCDHA13* | 58.3% (43.3-72.1%) |  | 97.1% (82.9-99.8%) |  | 3.68 |
| *SST* | 50.0% (35.4-64.6%) |  | 91.2% (75.2-97.7%) |  | 4.48 |
| *LOC100289333* | 79.2% (64.6-89.0%) |  | 85.3% (68.2-94.5%) |  | 6.20 |
| *BOLL* | 66.7% (51.5-79.2%) |  | 91.2% (75.2-97.7%) |  | 4.69 |
| *FAM19A4* | 70.8% (55.7-82.6%) |  | 79.4% (61.6-90.7%) |  | 6.47 |
| *MIR129-2* | 64.6% (49.4-77.4%) |  | 88.2% (71.6-96.2%) |  | 7.74 |
| *ZIC1* | 70.8% (55.7-82.6%) |  | 97.1% (82.9-99.8%) |  | 3.29 |
| *SOX1* | 68.8% (53.6-80.9%) |  | 94.1% (78.9-99.0%) |  | 5.25 |

**
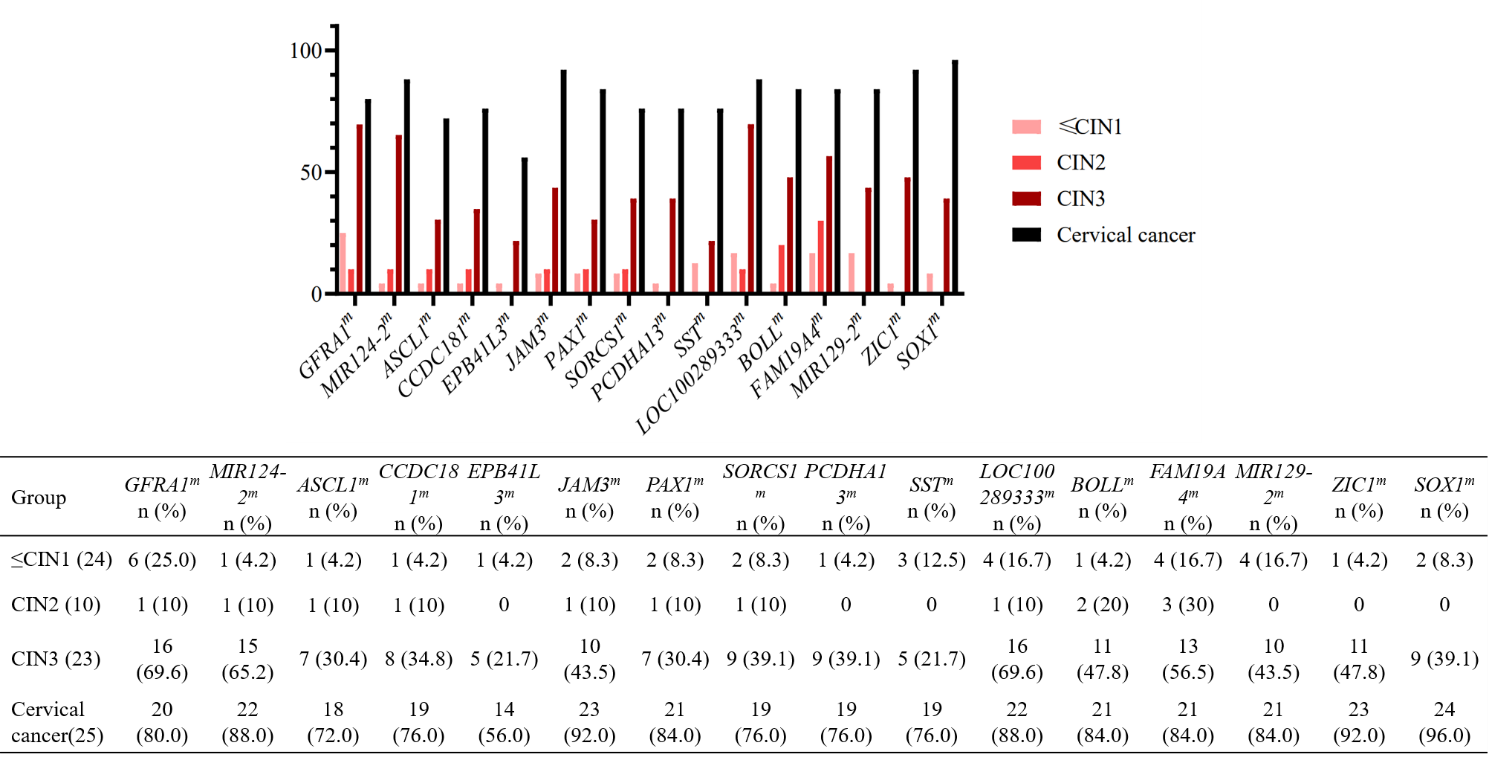
**

**Fig. S1. Sixteen genes methylation positivity in cervical scrapings (n = 82). The distribution of the methylation positivity of the 16 candidate markers in ≤CIN1, CIN2, CIN3, and CC group.**

**Table. S3. Sensitivity and specificity of cytology and DNA methylation for detecting CIN3+ in hrHPV-positive women.**

|  | sensitivity |  | specificity |  | AUC |
| --- | --- | --- | --- | --- | --- |
|  | 95% CI |  | 95% CI |  | 95% CI |
| Cytology | 66.7% (24.2-94.0%) |  | 9.1% (0.48-42.9%) |  | 0.379 (0.081-0.677) |
| Methylation markers^a^ | | | | | |
| *miR124-2^m^*  *JAM3^m^*  *LOC100289333^m^*  *ZIC1^m^*  *SOX1^m^* | 66.7% (24.2-94.0%)  33.3% (6.0-75.9%)  66.7% (24.2-94.0%)  66.7% (24.2-94.0%)  83.3% (36.5-99.1%) |  | 72.7% (39.3-92.7%)  100.0% (67.9-100%)  90.9% (57.1-99.5%)  90.9% (57.1-99.5%)  81.8% (47.8-96.8%) |  | 0.697 (0.423-0.971)  0.667 (0.369-0.964)  0.788 (0.533-1.000)  0.788 (0.533-1.000)  0.826 (0.603-1.000) |

^a^Using a threshold for positivity at a methylation ratio of 8.56 for *MIR124-2*, 5.94 for *JAM3*, 6.27 for *LOC100289333*, 3.29 for *ZIC1*, and 5.25 for *SOX1*. ^b^The performance of each marker in cervical scrapes was evaluated by AUC with 95% (CI).


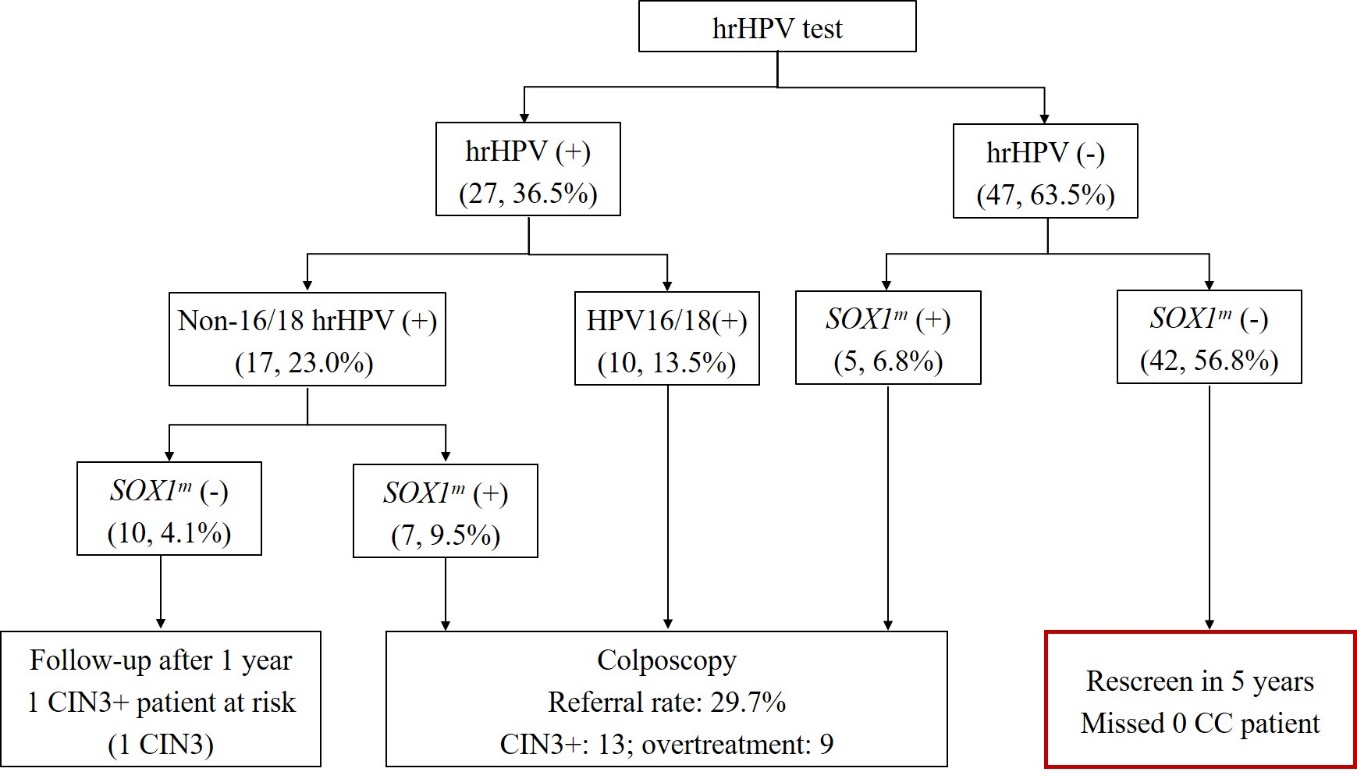


**Fig. S2 *SOX1^m^* analysis in the Chinese cohort**
